# SENP3 mediated DeSUMOylation of macrophage derived CCL17 accelerates atherosclerosis via regulation of Treg

**DOI:** 10.1007/s10565-025-10099-3

**Published:** 2025-11-21

**Authors:** Xiliang Zhao, Fenfang Zhang, Jianjun Du, Yaodong Ding, Yang Zhang, Yong Zeng, Yicong Ye

**Affiliations:** 1 Department of Cardiology, Beijing Anzhen Hospital, Capital Medical University, No.2, Anzhen Road Chaoyang District, Beijing, 100029 People’s Republic of China; 2 Department of Cardiology, Yangquan First People’s Hospital, Yangquan, 045000 People’s Republic of China; 3 Department of Cardiology, The First People’s Hospital of Keerqin District, Tongliao, 028000 People’s Republic of China

**Keywords:** Atherosclerosis, Lipid accumulation, Inflammation, Macrophages, CCL17

## Abstract

**Background:**

Atherosclerosis (AS) is a cardiovascular problem, which is featured by the accumulation of lipids in the intimal layer of the arterial wall and inflammatory reaction of immune cells. CCL17 is an inflammatory mediator associated with promoting AS. Nevertheless, the specific role of CCL17 and its upstream regulatory mechanisms in macrophage mediated inflammation and AS remain unclear.

**Methods:**

An AS mice model was established by subjecting ApoE^−/−^ mice to a high-fat diet (HFD). Constructing an AS cell model by treating primary macrophages with oxidized low-density lipoprotein (ox LDL). Injecting shRNA wrapped by AAV virus into the tail vein of mice knocked down CCL17 and SENP3 in mice. Hematoxylin–eosin (HE) and oil red O staining were used to detect arterial injury in mice. The changes of Treg cells were detected by flow sorting. Cycloheximide (CHX) and immunoprecipitation were used to detect the level of DeSUMOylation of CCL17 modified by SENP3.

**Results:**

The CCL17 and SENP3 expression in plaque sample of AS mice were significantly up-regulated. Knocking down CCL17 or SENP3 in mice could reverse the vascular damage, lipid accumulation, the increase of the blood lipid levels and the increase of inflammatory reaction in AS mice. On the molecular mechanism level, SENP3 increased the protein stability of CCL17 and thus increased CCL17 expression by DeSUMOylation modification at K115 site of CCL17 protein. In macrophages induced by oxLDL, CCL17 and CCL22 affect the chemotaxis of Treg competitively.

**Conclusion:**

This study showed that SENP3 mediated deSUMOylation of CCL17, increase CCL17 expression in macrophage. CCL17 secreted by macrophage regulating Treg recruitment through the competitive interaction between CCL17 and CCL22 and thus aggravated AS. Our findings provide a new regulatory mechanism and potential target for AS treatment.

**Graphical Abstract:**

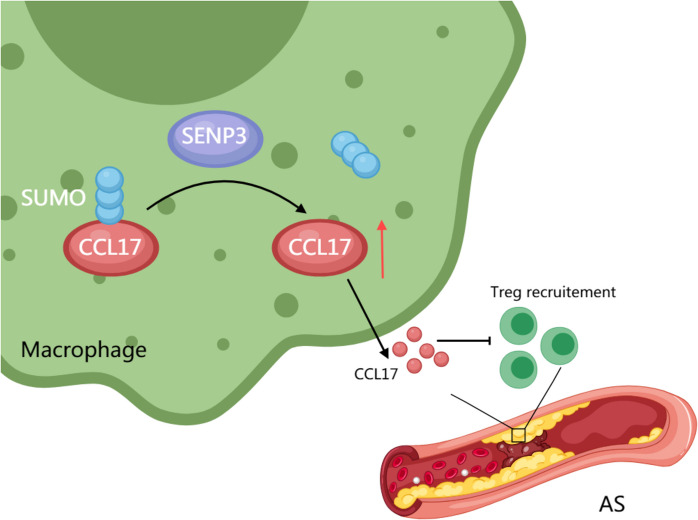

**Supplementary Information:**

The online version contains supplementary material available at 10.1007/s10565-025-10099-3.

## Highlights

1. CCL17 and SENP3 expression in plaque tissue of AS mice were increased.

2. Knocking down CCL17 or SENP3 improved the progress of AS.

3. The stability of CCL17 protein is regulated by SENP3-mediated DeSUMOylation.

4. Macrophages stimulated by ox-LDL affect the chemotaxis of Treg through CCL17 and CCL22 competition.

**Supplementary Information:**

The online version contains supplementary material available at 10.1007/s10565-025-10099-3.

## Introduction

Cardiovascular related diseases are a significant cause of disease-related deaths and disabilities worldwide, especially in China (Inzucchi et al. 2022). The pathological factors of cardiovascular diseases include unhealthy diet and lifestyle, such as smoking and high-fat food. Atherosclerosis (AS) is the primary pathological change during cardiovascular diseases, and AS-related cardiovascular disease causes half of the cardiovascular disease-related death (Cohen Tervaert 2013; Hannawi et al. 2020). Research have revealed that the AS pathology involves the thrombosis formation, chronic inflammation, oxidative stress, lipid infiltration, autophagy, and endothelial injury (Tabares-Guevara et al. 2021). Nowadays, it is well-recognized that inflammatory response functions throughout the development of AS (Pedro-Botet et al. 2020). The inflammation and accumulation of lipid in macrophages are regarded as the hallmarks of AS (Pedro-Botet et al. 2020). The increased accumulation of cholesterol and lipid and the elevated levels of inflammatory factors in blood promote the inflammation in blood vessel (Zhu et al. 2018). During AS, macrophages transform into foam cells upon intake of oxidized low-density lipoprotein (ox-LDL), which then releases excessive inflammatory factors (Libby 2021). Besides, upon vascular injury, the production of adhesion molecules is enhanced (Libby 2021).

Chemokines are a family of cytokines that exert their biological effects by interacting with G protein-coupled receptors (GPCRs) to initiate intracellular signaling cascades and promote migration of cells to chemokine sources (van der Vorst et al. 2015; Vilgelm and Richmond 2019). Various studies have implied the significance of chemokines in the progression of AS (Bakogiannis et al. 2019; Gencer et al. 2021). C–C chemokine ligand 17 (CCL17) is a chemokine and the downstream of GM-CSF to mediate the inflammation in macrophages and monocyte (Conaghan et al. 2019; Hamilton 2019). It has been reported that blockade of CCL17 could ameliorate GM-CSF-induced inflammatory pain in arthritic disease (Lee et al. 2020). An interesting study found that CCL17 derived from dendritic cells accumulated in pathological tissues in AS mice, and accelerated the process of atherosclerosis by regulating the steady state of Tregs (Weber et al. 2011). However, the role of macrophage-secreted CCL17 in promoting AS progression and its underlying molecular regulatory mechanisms remain unclear.

SUMOylation is a post-translational modification process mainly mediated by small ubiquitin-like modifiers (SUMOs) (Talamillo et al. 2020). SUMO protein was originally expressed in a precursor/inactive form, and Sentrin/SUMO-specific protease (SENPs) can be converted into a mature SUMO protein with catalytic activity by C- terminal cleavage (Kumar and Zhang 2015). At present, the main SENP family proteins include SENP1-6, among which SENP1, SENP2, SENP3 and SENP5 are the most common (Witty et al. 2010). It has been reported that SENP3 is increased in AS, and it can stabilize β-catenin protein by removing SUMOylation and promote the migration of VMSC, thus accelerating the progress of AS (Cai et al. 2021). At the same time, SENP3 has been proved to regulate the macrophage polarization, SENP3 increases the number of M1 macrophages, while inhibiting SENP3 can promote the polarization of M2 macrophages (He et al. 2023). Our findings reveal that CCL17 possesses a highly reliable SUMOylation site and a SUMO-interacting motif as identified through the SUMOylation database. Based on this, we hypothesize that during the progression of AS, SENP3-mediated de-SUMOylation enhances the stability of the CCL17 protein, leading to increased release of CCL17 by macrophages.

We hypothesized that SENP3 promotes AS progression by mediating the de-SUMOylation of CCL17, thereby enhancing its stability and expression and increasing its release from macrophages. This hypothesis was validated using AS mouse models and cell-based experiments. To further investigate, the supernatant from macrophages was co-cultured with Treg cells, revealing that macrophage-secreted CCL17 and CCL22 competitively influence Treg chemotaxis. Our study provides an in-depth analysis of the interaction between macrophages and Treg cells in AS, offering a theoretical foundation for the development of immunotherapeutic strategies targeting AS.

## Materials and methods

### Materials

Plasmid of ShRNAs targeting CCL17 (shCCL17), Plasmid of shRNA targeting SENP3 (shSENP3) and negative control (shNC), WT and K163R mutants CCL17 overexpression vectors were purchased from Transheep (Shanghai, China). Briefly, K163R point mutation was introduced by site-directed mutagenesis using mutagenic primers and DNA polymerase. After PCR, samples were digested with DpnI to eliminate the template plasmid and transformed into DH5α competent cells. Positive clones were verified by Sanger sequencing. The autophagy inhibitor 3-Methyladenine (3MA; HY-19312) and AKT activator SC79 (HY-18749) were bought from MCE (MA, USA). Ox-LDL was bought from Shanghai Yeasen. Triglyceride (TG) Colorimetric Assay Kit and Low-Density Lipoprotein Cholesterol (LDL-C) Colorimetric Assay Kit were bought from elabscience (Wuhan, China). Cholesterol/Cholesteryl Ester Assay Kit (ab102515) was bought from Abcam (USA).

### Transfection vector packaging

Adeno-associated virus (AAV) for in vivo knockdown were also purchased from rom Transheep (Shanghai, China). Briefly, AAV-shRNA vectors were generated by cloning shRNA sequences into a transfer plasmid HEK293T or macrophages cells were co-transfected with the plasmid, a Rep-Cap packaging plasmid, and a helper plasmid using PEI. After transfection for 48 h, the virus particles were collected and purified via iodixanol gradient ultracentrifugation, and quantified by qPCR to determine viral genome titers.

### Animals

Male C57BL/6 J mice and ApoE^−/−^mice aged 6 to 8 weeks and weighted around 20 g were purchased from Hunan SJA laboratory animal company (Changsha, China) and maintained in a special pathogen-free (SPF) environment and 12/12 dark/light cycle. Mice in indicated group received a single tail vein injection of 3 × 10^11^ VG AAV-sh-CCL17 or AAV-sh-SENP3 virus or AAV-sh-NC. Immediately after AAV injection, mice were subjected to establish the atherosclerosis model, the ApoE^−/−^ mice were fed with a high-fat diet (HFD) (Hunan SJA laboratory animal company) for three months continually. The control group was fed with common feed. After the model was established, 1 mL serum was collected from each mouse. Mice were euthanized for spinal dislocation and the relevant tissue samples were collected for subsequent detection. For surgical procedures, mice were anesthetized with 2 ~ 3% isofluranein oxygen. All experiments were approved by the Beijing Anzhen Hospital Ethics Committee.

### Histopathological staining

For Hematoxylin–eosin (HE) staining, the fixed mouse aorta tissue was dehydrated, transparent and embedded into paraffin Sects. (5 μm) for later use. After dewaxing and rehydrating, HE staining, dehydration and sealing, results were recorded under a microscope. Image J software measures the relative area of plaque, that is, the ratio of plaque area to lumen area.

For oil red O staining, mouse aorta tissue was dehydrated at room temperature and put into oil red O dye. After 10 min, the sample was taken out and rinsed in 85% isopropanol. Finally, the sections were fixed and the staining results were observed and recorded.

The mice heart aorta was sliced and immunofluorescence experiment was carried out. According to the steps of antigen repair-blocking-primary antibody incubation-secondary antibody incubation -DAPI staining, the immunofluorescence experiment was carried out. Finally, the section was placed on a glass slide, seal it with glycerol, and take a photo to record. Primary antibody: anti-CCL17 (ab182793, 1:50, Abcam); anti-CD25 (ab215206, 1:50, Abcam); anti-SENP3 (17,659–1-AP, 1:250, Proteintech); anti-CD68 (ab283654, 1:50, Abcam).

### Cell culture and treatments

Mouse macrophage Mouse Bone Marrow-Derived Macrophage Cells were brought from the Procell (Wuhan, China) and were maintained in DMEM (Gibco, USA) that complemented with 10% fetal bovine serum (Thermo, USA) and 100 U/ml penicillin and 100 μg/ml streptomycin (Gibco, USA). Cell culture was performed in a humidified 37 °C incubator that filled with 5% CO_2_.Transfection of primary macrophage macrophages with lentiviral plasmid carrying shRNA. The cells were induced 24 h after transfection. To induce foamy macrophages, the primary macrophage cells were pre-stimulated with DMEM medium (without serum) for 12 h, treated with indicated drugs, then incubated with DMEM + 10% FBS + 75 μg/mL ox-LDL for 24 h. In some case, 30 μM 2-D08 (Thermo) to the culture medium and incubate with cells for 48 h. Naïve CD4⁺ T cells were isolated from mouse spleens using a CD4⁺ T cell isolation kit (Miltenyi Biotec) via magnetic-activated cell sorting. Cells were cultured in RPMI 1640 medium (Gibco) supplemented with 10% FBS, 100 U/mL penicillin/streptomycin, 50 μM β-mercaptoethanol, recombinant mouse IL-2 (10 ng/mL, MCE), and recombinant TGF-β1 (5 ng/mL, MCE). For T cell receptor stimulation, culture plates were pre-coated with anti-CD3 antibody (2 μg/mL, Abcam) and supplemented with soluble anti-CD28 antibody (2 μg/mL, Abcam). Cells were incubated for 5 days to allow differentiation into induced Tregs (iTregs). The efficiency of Treg induction was confirmed by FOXP3 expression using flow cytometry. Prior to the transwell migration assay, the induced Tregs were treated with recombinant mouse CCL17 (MCE, HY-P71891A) or recombinant mouse CCL22 (MCE, HY-P7248) or anti-CCL17 (R&D, Catalog #: MAB529) and anti-CCL22 antibodies (R&D, Catalog #: MAB529).

### Lipid assay

Aortic tissues and primary macrophage cells were washed with ice-cold PBS and were lysed with RIPA lysis buffer. The levels of total cholesterol (TC) were measured by Cholesterol/Cholesteryl Ester Assay Kit. Triglyceride (TG) and low-density lipoprotein cholesterol (LDL-C) were measured by using the commercial ELISA kits according to manufacturer’s introduction. Briefly, samples were incubated with indicated antibodies that pre-coated in 96-well plates. The absorbance at 450 nm was detected and recorded.

### Western blot analysis

The aortic tissues and primary macrophage cells were split with RIPA buffer (Beyotime, China) and amounts of proteins was quantified with BCA kit (SolarBio, China). 35 µg proteins were separated with SDS-PAGE gel and were transferred to PVDF membranes. The blots were incubated with 5% non-fat milk at room temperature and were probed with primary antibodies that target anti-CCL17 (Proteintech, 22,342–1-AP, 1:1000), anti-SENP3 (Proteintech, 17,659–1-AP, 1:1000) and anti-β-actin (Proteintech, 66,009–1-Ig, 1:5000) overnight at 4 °C. Next day, the films were treated with secondary antibodies (1:10,000). The protein bands were visualized after reaction with ECL reagent (Millipore, Germany). Internal reference protein: β-actin. Secondary antibodies were bought from Proteintech (China).

### Quantitative real-time PCR

Total RNA was extracted with TRIzol reagent (Invitrogen, USA) according to the manufacturer’s instructions. RNA concentration and purity were determined spectrophotometrically. Subsequently, 1 µg of total RNA was reverse transcribed into cDNA using the PrimeScript RT Reagent Kit (TaKaRa, Japan). Quantitative real-time PCR was performed, and relative gene expression levels were analyzed using the 2^−ΔΔCt^ method.

### Flow cytometry

The heart artery tissue of mice was ground and collected it by centrifugation. The cells were resuspended with 10 mL PBS, placed on Ficoll and centrifuged at room temperature of 1500 r/min for 20 min. Cells were equally divided into test tubes, washed once in PBS, and then resuspended to 10^6^ cells/mL. Fluorescein isothiocyanate (FITC) combined with CD4 (100,405, 1: 200, Biolegend) or CD25 combined with FOXP3 (364,703/154202, 1:100, Biolegend) was incubated at 4℃ for 20 min for staining. BD FACS-Calibur flow cytometer (BD-biosciences, USA) was used for analysis, and CD4 + cells were collected by gating.

### Detection of inflammatory factors by ELISA

According to the instructions of ELISA kit, the standard was diluted in gradient. After determining the optimum working solution concentration, the samples were treated as required and incubated with enzyme step by step. After color development, add the termination solution and measure the absorbance (OD, 450 nm) in a microplate reader. The ELISA kit: Mouse IL-1β antibody pair-BSA and Azide free (ab241673, abcam); Mouse IL-6 ELISA Kit (ab222503, Abcam).

### CHX protein chase

1 × 10^5^ primary macrophages were inoculated into 6-well plate and incubated with CHX of 150 μg/mL overnight. Collecting cell samples at 0, 2, 4 and 6 h respectively and subjected to western blot analysis.

### Immunoprecipitation (IP)

The cells to be detected were lysed with 250 μL RIPA buffer (Beyotime, China) and centrifuged to collect supernatant. 500 μg protein and A/G beads (Santa Cruz Biotechnology) were allowed to react at 4 C for 1 h. Centrifuge for 10 min and then collect the supernatant. Then, an equal amount of anti-CCL17 (ab182793, 1:50, abcam) was added to each supernatant, and incubated 12 h (4℃). Next, after adding an equal amount of A/G beads to each sample and incubating for 2 h, samples were collected and mixed with non-reducing sample loading buffer (39,001, Thermo Fisher Scientific). SUMO1/2, ubiquitin and CCL17 proteins were detected by western blot.

### Immunocoprecipitation (Co-IP)

After lysing the cells, the precipitate was collected by centrifugation. The supernatant was blended with GFP-Nanoab-Agarose (AB_2631357, Proteintech, Wuhan, China) overnight at 4℃. Then blended with Dynabeads protein G beads (10003D, Thermo Fisher Scientific, USA) at 4℃ overnight. Finally, SENP3 (17,659–1-AP, Proteintech) and CCL17 (ab182793, abcam) were detected by western blot.

### GST Pull-down

GST or GST–CCL17 fusion protein (100 µg) was immobilized on 50 µL glutathione–agarose beads at 4 °C for 1 h. After washing with binding buffer (20 mM Tris–HCl, 150 mM NaCl, 0.1% NP-40, 1 mM DTT), beads were incubated with FLAG–SENP3 fusion protein (100 µg) overnight at 4 °C with gentle rotation. Beads were washed extensively, and bound proteins were eluted by boiling in SDS sample buffer, then analyzed by western blot. GST alone was used as a negative control.

### Statistical analysis

Data in this study was shown as mean ± SEM of three independent experiments. Data analysis was conducted using a Graphpad Prism 9.0 software (Boston, MA, USA). Prior to statistical testing, data were assessed for normality using the Shapiro–Wilk test. Comparisons between two groups were conducted using the unpaired Student’s t-test. For comparisons among multiple groups, one-way ANOVA was used followed by Tukey’s post hoc test to determine significant differences between group means. A p-value of less than 0.05 was considered statistically significant.

## Results

### CCL17 was elevated in atherosclerotic model

**Fig. 1:**
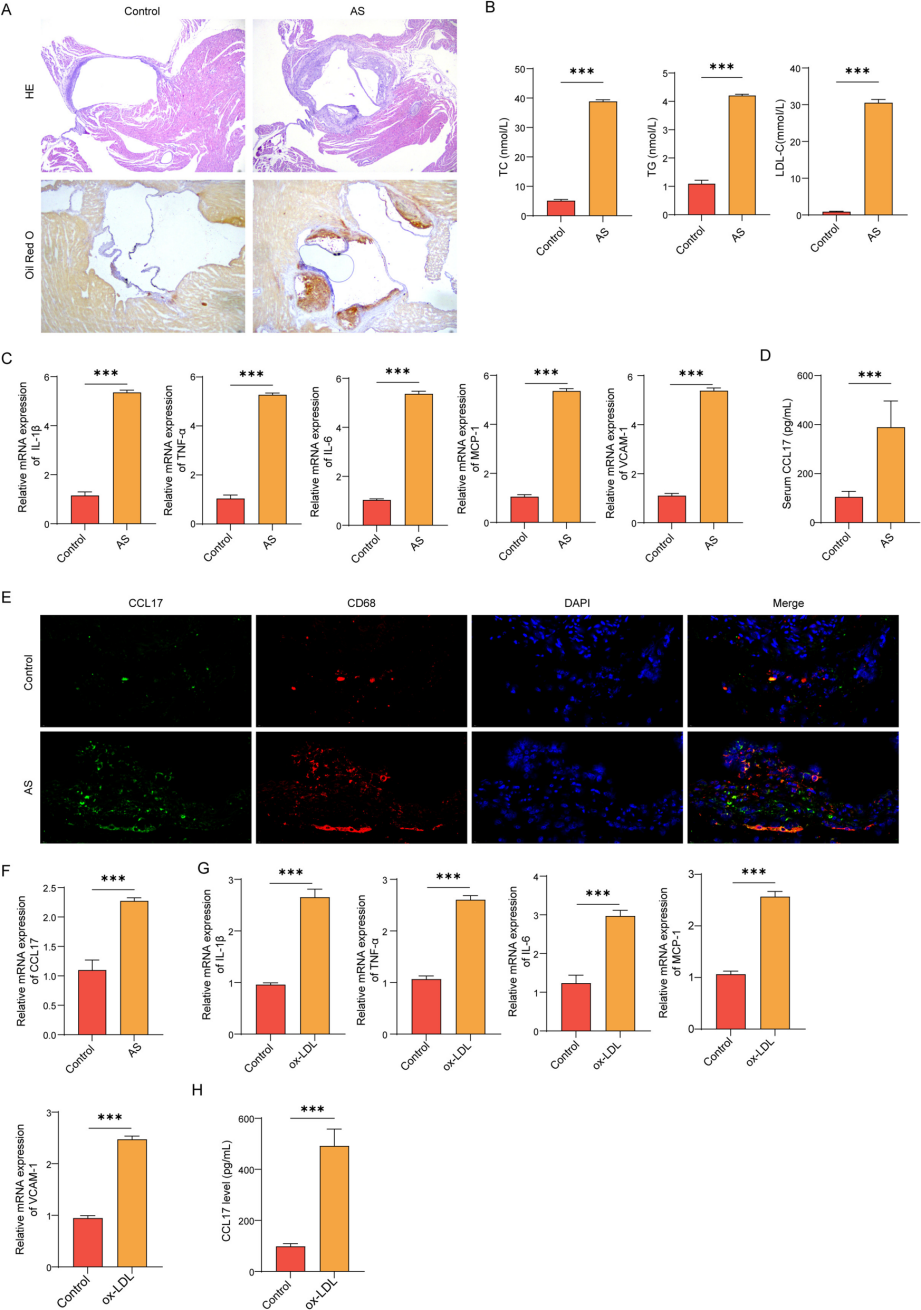
CCL17 was elevated in atherosclerotic model. The murine AS model was established by high-fat diet (HFD) to ApoE mice. **A** HE and Oil Red O analysis of aorta root tissues. **B** The levels of total cholesterol (TC), triglyceride (TG) and low-density lipoprotein cholesterol (LDL-C) in serum were measured by ELISA. **C** The (**C**) RNA levels of pro-inflammatory factors VCAM-1, MCP-1, TNF-α, IL-1β, and IL-6 was detected by qPCR assay. **D** Serum CCL17 level was detected by ELISA. **E** Immunofluorescence detection of co-localization of CCL17 and macrophages in plaque tissue of AS mice. **F** The level of CCL17 mRNA in mice plaque tissue was detected by qPCR. In vitro AS model was established by stimulating primary macrophage macrophages with ox-LDL. **G** The mRNA levels of inflammatory factors (IL-1β and IL-6) were detected by qPCR. **H** The level of CCL17 in cell supernatant was detected by ELISA. **I** The level of CCL17 in macrophages was detected by qPCR. *p < 0.05, *** *p* < 0.01, *** *p* < 0.001. *N* = 6

We first established the mouse AS model by high-fat diet (HFD) to AS mice. The results showed that the aortic intima of the control group mice was intact (Fig. 1 A). However, in mice from AS mice, the intima in aortic root was thickened, the structure of endothelial cells was incomplete (Fig. 1 A), and lipid accumulation increased in AS mice (Fig. 1 A). Similarly, the blood lipid levels (TC, TG and LDL-C) in the serum samples of AS mice were elevated (Fig. 1B). Besides, the mRNA expression of pro-inflammatory factors, including the VCAM-1, MCP-1, TNF-α, IL-1β, and IL-6 were remarkably higher in the aortic tissues of AS mice (Fig. 1 C). Noteworthy, the content of CCL17 protein in serum of AS mice increased significantly (Fig. 1D). Then, immunofluorescence co-localization showed that CCL17 was mainly expressed in macrophages (Fig. 1E). Similarly, the expression of CCL17 in plaque tissue of AS mice was also up-regulated (Fig. 1 F). Next, we constructed an in vitro model of primary macrophage cells stimulated by ox-LDL. In macrophages induced by ox-LDL, the mRNA levels of inflammatory cytokine were significantly increased (Fig. 1G), suggesting an inflammatory reaction. Further detection of CCL17 expression in cell supernatant and macrophages displayed that CCL17 in macrophage supernatant and cells increased after ox-LDL stimulation (Fig. 1H and 1I).

### Silencing CCL17 improves atherosclerosis in mice

**Fig. 2:**
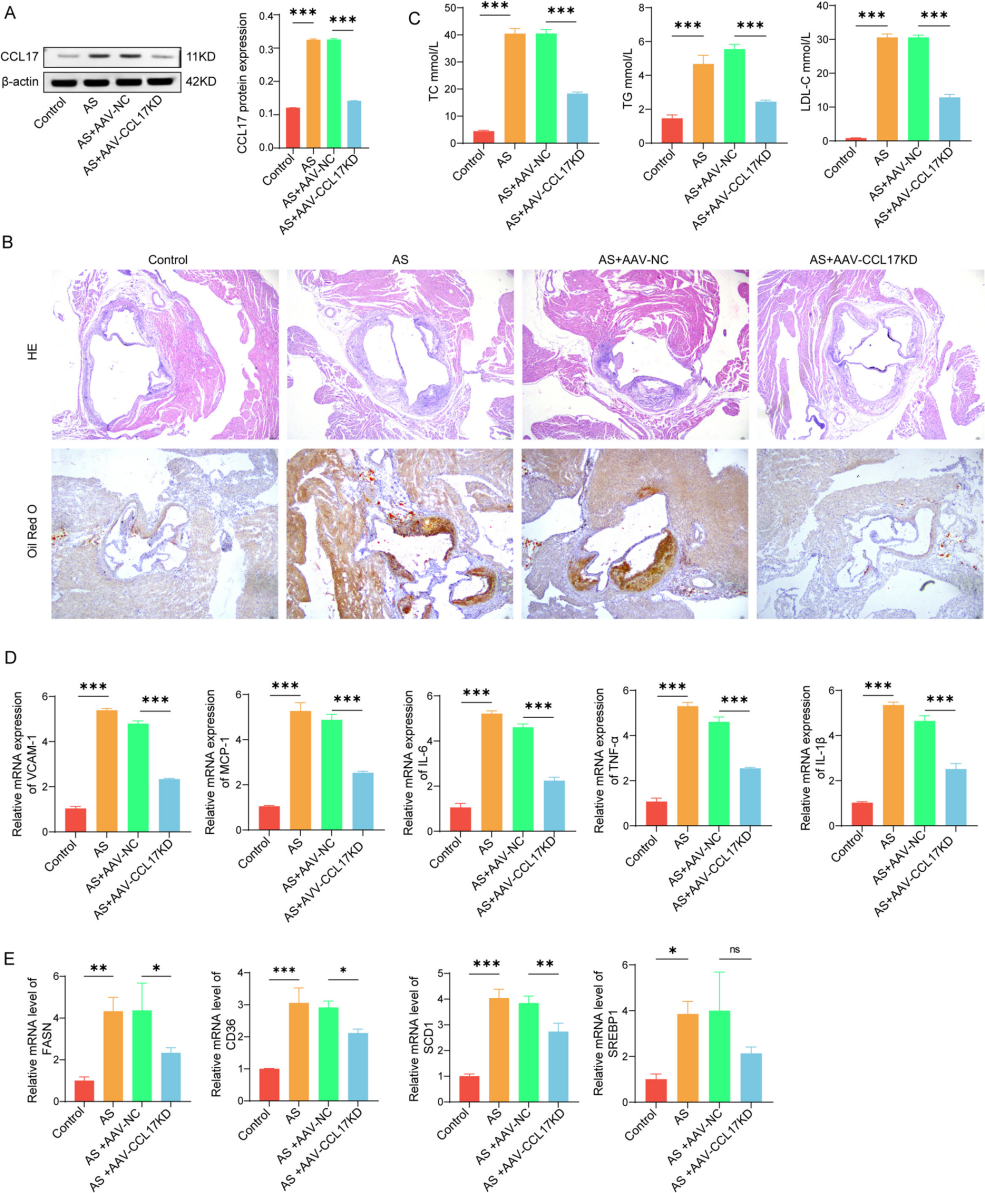
Silencing CCL17 improves atherosclerosis in mice. After 4 weeks of AS modeling, sh-CCL17 encapsulated by AAV virus was injected into the tail vein of mice to knock down the expression of CCL17 in mice. **A** Western blot was used to detect the protein expression of CCL17 in mice plaque. **B** HE and oil red O staining were used to observe the pathological changes of arterial tissue in mice. **C** The levels of TC, TG and LDL-C in AS mice were measured by ELISA. **D** qPCR was used to detect the mRNA level of inflammatory factors (IL-1β, and IL-6). **E** The expression of lipid genes FASN, CD36, SCD1 and SREBP1 was detected by qPCR. * *p* < 0.05, *** *p* < 0.01, *** *p* < 0.001. *N* = 6

We knocked down the expression of CCL17 by injecting CCL17 shRNA wrapped by AAV virus into the tail vein of mice, and further verified the effect of CCL17 on AS. AAV-CCL17 reduced the elevated CCL17 level in plaque of AS mice (Fig. 2 A). Histological analysis of aortic root showed that knocking down CCL17 could reduce the tissue damage and lipid accumulation in AS mice (Fig. 2B). In addition, inhibition of CCL17 can inhibit the blood lipid levels in AS mice (Fig. 2 C), and inhibit the production of proinflammatory factors (Fig. 2D). Further measurement of lipid genes, namely FASN, CD36, SCD1 and SREBP1, showed that reducing the expression of CCL17 could inhibit the transcription level of these genes in AS mice (Fig. 2E). These data show that the knock-out of CCL17 reduces the progress of AS in vivo.

### Silencing CCL17 promotes the recruitment of Treg in AS plaque tissue

**Fig. 3:**
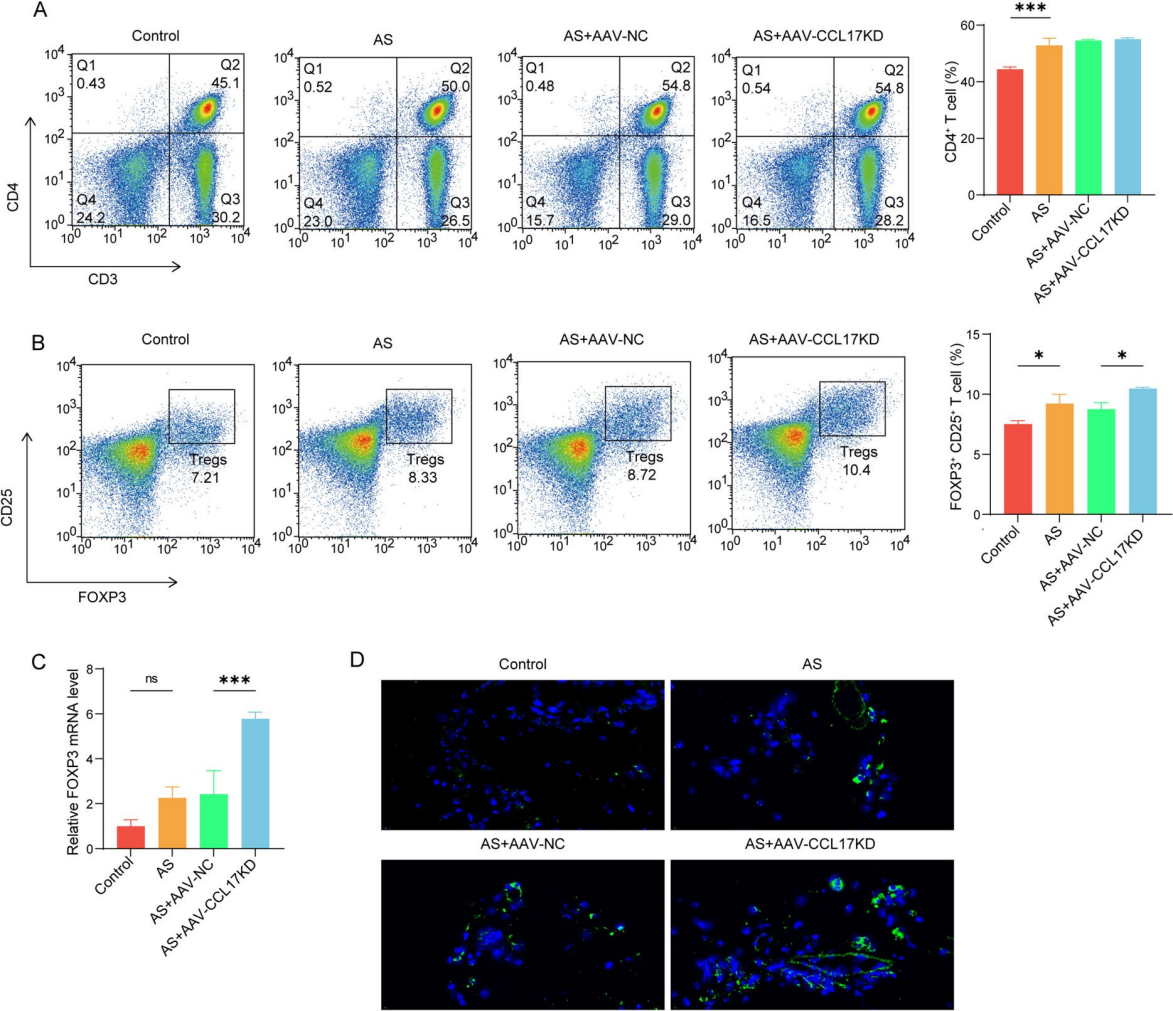
Silencing CCL17 promotes the recruitment of Treg in AS plaque tissue. **A** The total CD4+ cells (CD3, CD4) in plaque tissue were detected by flow cytometry, and the proportion of cells in the upper right quadrant was counted. **B** The proportion of Treg cells (FOXP3, CD25) in CD4 cells was detected by flow cytometry. **C** The expression of FOXP3 in mouse plaque was detected by qPCR. **D** The expression of CD25 in mouse plaque was detected by immunofluorescence. *p<0.05, **p<0.01, ***p<0.001. *N*=6

Furthermore, we detected the changes of Treg cell enrichment in plaque tissue of AS mice after CCL17 was knocked down. CD4 + cells (CD3, CD4) were abundant in plaque tissue of AS mice, but the number of CD4 + cells did not change significantly after CCL17 was knocked out (Fig. 3 A). Then, we detected the proportion of Treg cells (FOXP3, CD25) in CD4 cells, and found that Treg cells increased greatly after CCL17 was knocked out (Fig. 3B). The FOXP3 mRNA was up-regulated in the plaque tissue of AS mice, and it increased significantly after CCL17 was knocked out (Fig. 3 C). Additionally, immunofluorescence revealed that CD25 expression was further increased by CCL17 inhibition (Fig. 3D). These results indicated that silencing CCL17 promote recruitment of Treg in AS.

### Macrophages stimulated by oxLDL affect the chemotaxis of Treg through CCL17 and CCL22 competition

**Fig. 4:**
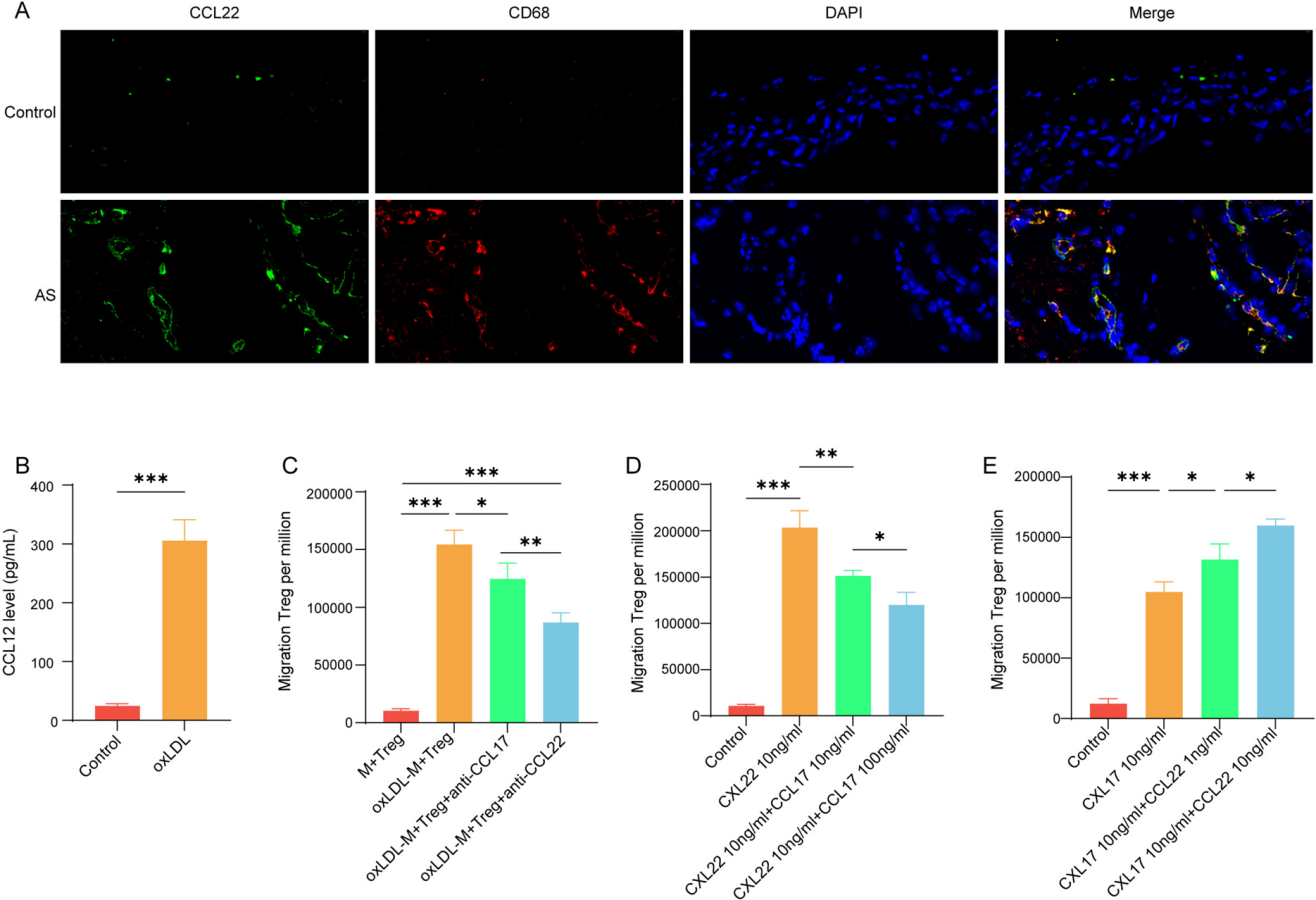
Macrophages stimulated by oxLDL affect the chemotaxis of Treg through CCL17 and CCL22 competition. Macrophages were stimulated with oxLDL, and Treg cells were co-cultured with macrophage supernatant. **A** Immunofluorescence detection of co-localization of CCL22 and macrophages (CD68) in plaque tissue of AS mice. **B** The content of CCL22 in the supernatant of macrophages was detected by ELISA. **C** Transwell detected the effect of macrophages on the chemotaxis of Treg. (**D**-**E**) Transwell detected the migration of Treg cells. * *p* < 0.05, ** *p* < 0.01, *** *p* < 0.001. *N* = 3

Next, we investigated the mechanism by which CCL17 knockout leads to increased Treg recruitment. In the aforementioned study, the researchers demonstrated that CCL17 does not affect Treg differentiation or proliferation, but rather modulates their chemotaxis by competitively binding to CCR4 on Tregs, interfering with CCL22—a chemokine with stronger chemotactic potency. To validate this mechanism in our model, we first examined whether CCL22 levels were elevated in atherosclerosis. As shown in Fig. 4 A, similar to CCL17, CCL22 was upregulated in the AS model and colocalized with CD68-positive macrophages. Similarly, the content of CCL22 in the supernatant of macrophages was detected, and it was found that the level of CCL22 increased after oxLDL stimulation (Fig. 4B). We assessed the chemotactic response of Tregs and found that macrophage-conditioned supernatant promoted Treg migration. Neutralization of either CCL17 or CCL22 with specific antibodies significantly reduced Treg migration, with CCL22 blockade exhibiting a more pronounced inhibitory effect. (Fig. 4 C). Interestingly, we also found that CCL17 can inhibit the Treg chemotaxis induced by CCL22 (Fig. 4D). And increasing the concentration of CCL22 can enhance the chemotaxis of Treg induced by CCL22 (Fig. 4E). Furthermore, we also found that inhibiting CCL17 had no observably interfere with CCL22 expression (Fig. S2). This result is consistent with previous studies, suggesting that CCL17 and CCL22 compete to induce Treg chemotaxis in AS model, and CCL22 has stronger chemotaxis ability.

### The stability and expression of CCL17 protein is regulated by SENP3-mediated DeSUMOylation

**Fig. 5:**
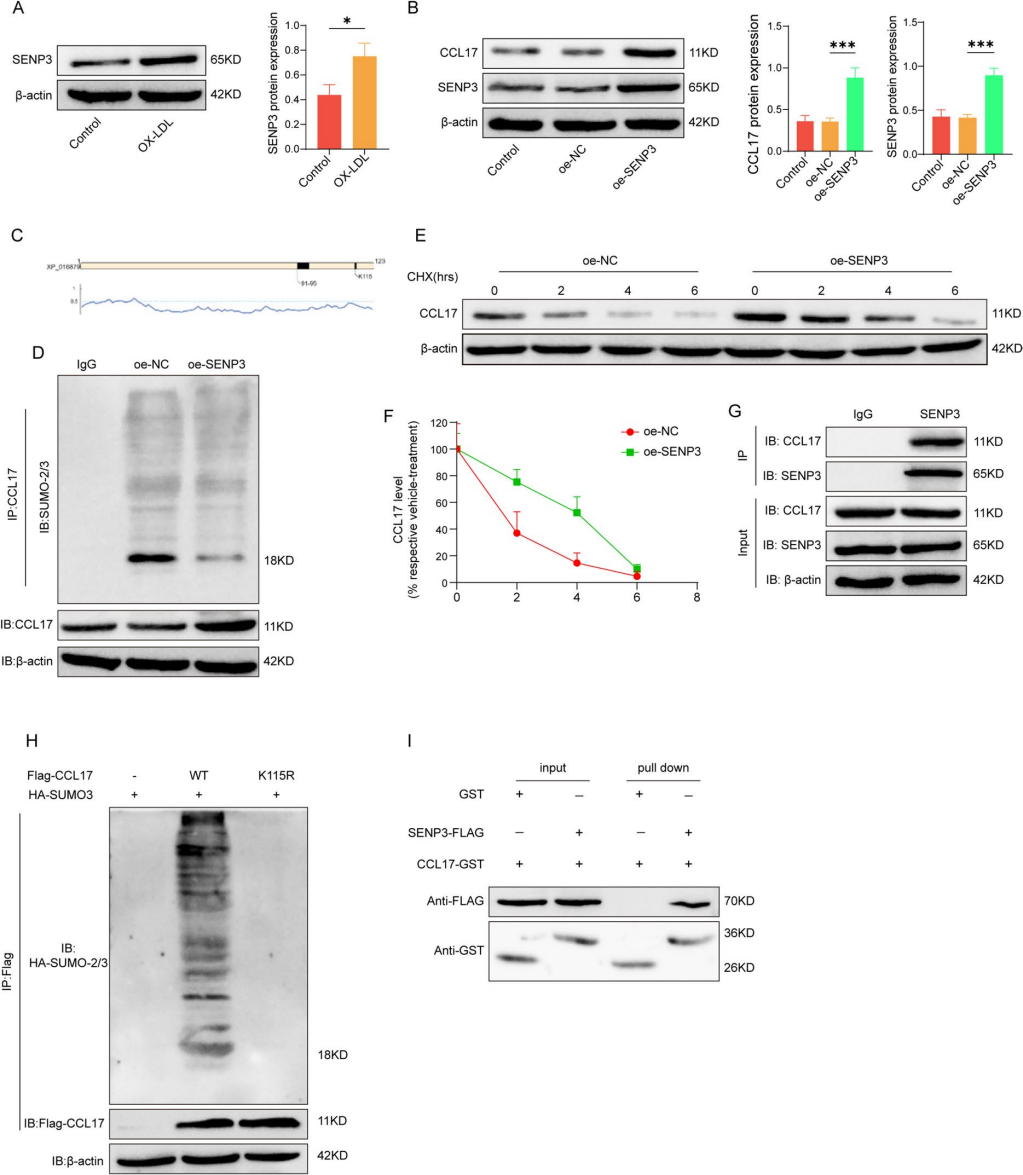
The stability of CCL17 protein is regulated by SENP3-mediated de-SUMOylation. **A** Western blot detected the level of SENP3 in macrophages induced by oxLDL. **B** SENP3 was overexpressed in macrophages, and the expressions of SENP3 and CCL17 were detected by western blot. **C** Bioinformatics on-line prediction of sumoylation site of CCL17 protein. **D** IP detection of the effect of SENP3 on the DeSUMOylation of CCL17. **E** & (**F**) The effect of SENP3 on the stability of CCL17 protein was detected by CHX protein degradation experiment. **G** Co-IP experiment verified the binding relationship between SENP3 and CCL17. **H** The DeSUMOylation of mutant CCL17 of K115R was detected by IP. **I** The binding relationship between SENP3 and CCL17 was verified through GST Pull-down. * *p* < 0.05, ** *p* < 0.01, *** *p* < 0.001. *N* = 3

SENP3 has been shown to be elevated in atherosclerosis, where it stabilizes β-catenin protein through desumoylation modification, promoting VMSC migration and exacerbating atherosclerosis(Cai et al. 2021). Additionally, SENP3 has been found to promote the polarization of pro-inflammatory M1 macrophages, while inhibiting SENP3 can promote the polarization of anti-inflammatory M2 macrophages(Xiao et al. 2022). Bioinformatics prediction using the SUMOylation website indicates that CCL17 has high-confidence SUMOylation sites and SUMO-binding protein regions. oxLDL induced macrophages, SENP3 expression in macrophages also increased significantly (Fig. 5 A). After transfection of SENP3 overexpression vector in macrophages, the protein levels of SENP3 and CCL17 increased (Fig. 5B). For the sake of verify the possible molecular mechanism, we predicted the SUMOylation site of CCL17 protein through biological information, suggesting that 91–95 sites of CCL17 protein are sumo enzyme binding sites. K115 is the sumo modification site (Fig. 5 C). Furthermore, the influence of SENP3 on the DeSUMOylation of CCL17 was analyzed, and it was confirmed that SENP3 could DeSUMOylation modify CCL17 protein (Fig. 5D). We also found that overexpression of SENP3 can promote the stability of CCL17 protein (Fig. 5E and 5 F). Finally, it was confirmed that SENP3 and CCL17 protein have direct binding effect (Fig. 5G). Interestingly, we constructed the FLAG-CCL17 plasmid of K115R mutant. The results showed that no DeSUMOylation was detected in CCL17 after K115R mutation, which confirmed that K115 was the main DeSUMOylation site of CCL17 (Fig. 5H). At the same time, GST Pull-down was conducted to verify the direct binding relationship between SENP3 and CCL17, and it was found that SENP3 and CCL17 have a direct binding effect (Fig. 5I). These results suggest that SENP3 can modify CCL17 protein by DeSUMOylation, thus affecting the stability and expression of CCL17.

### Knocking down SENP3 improved the progress of AS

**Fig. 6:**
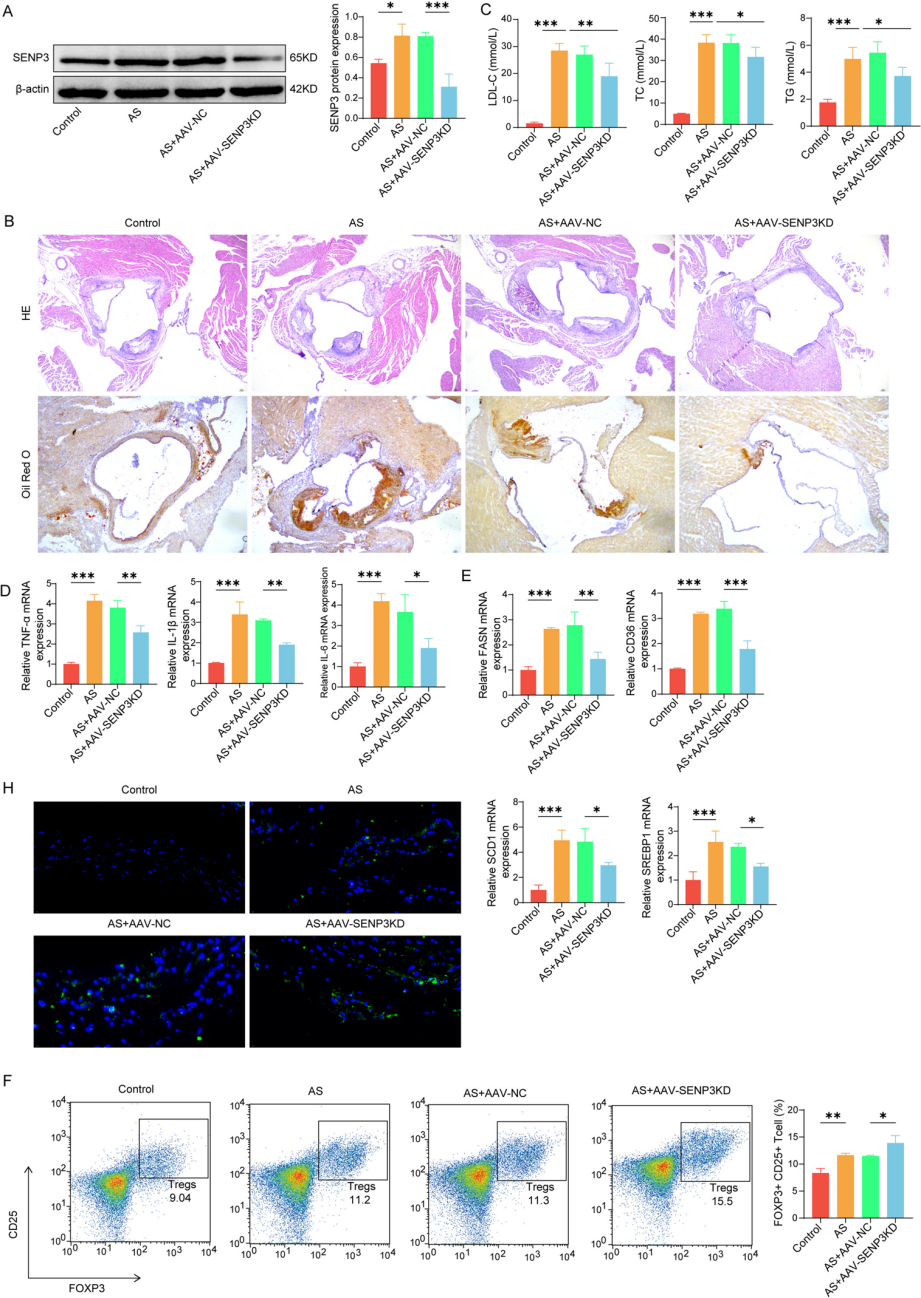

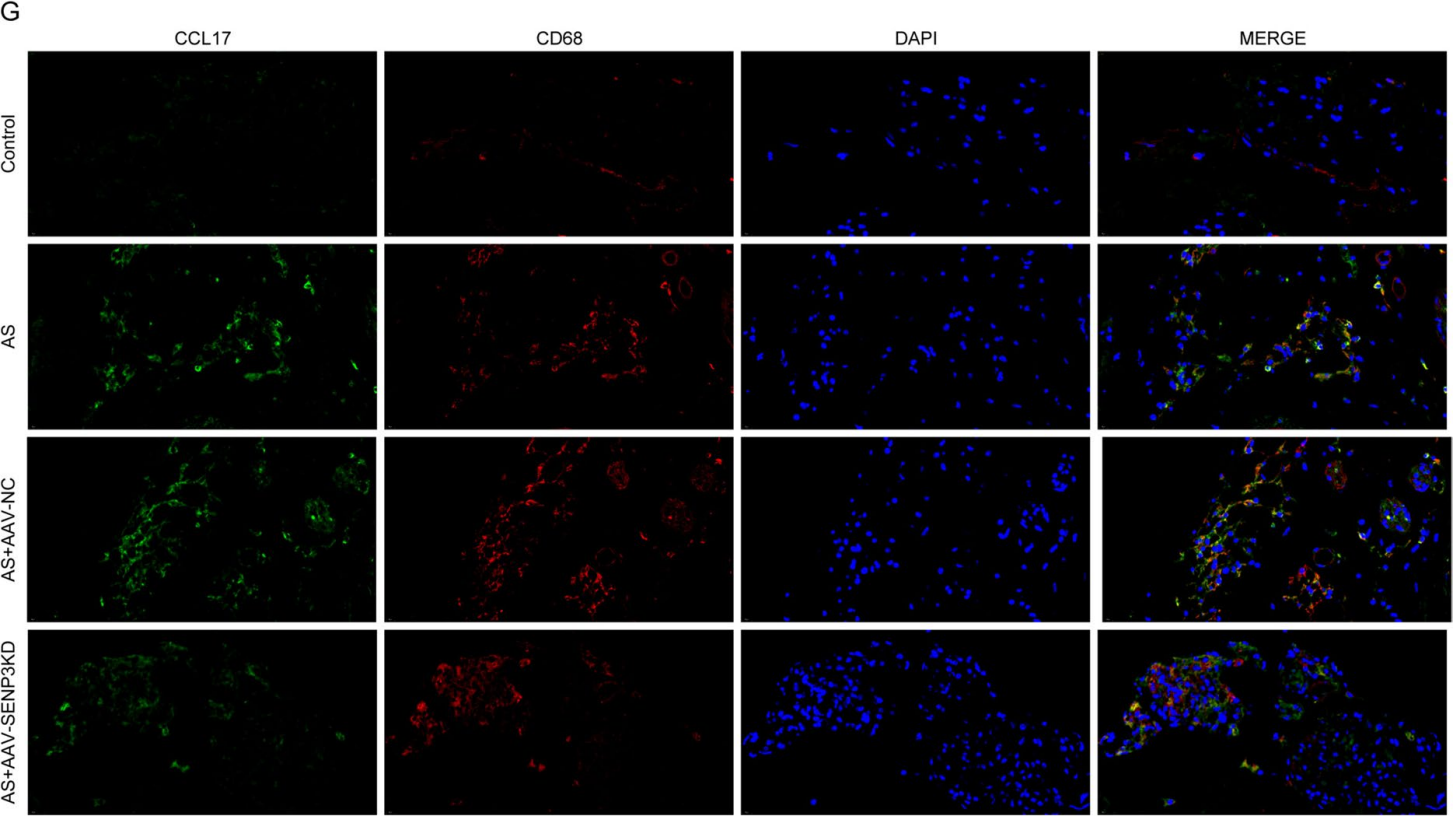
Knocking down SENP3 improved the progress of AS. After 4 weeks of AS modeling, sh-SENP3 encapsulated by AAV virus was injected into the tail vein of mice to knock down the level of SENP3 in mice. **A** Western blot detected the expression of SENP3 in mice plaque tissue. **B** HE staining and oil red O staining were used to observe the pathological changes of arterial tissue in mice. **C** The contents of TC, TG and LDL-C in mice were detected by ELISA. **D** The levels of inflammatory factors in mice were detected by ELISA. **E** qPCR detected the expression level of lipid-related genes. **F** Flow detection of Treg cells in mice plaque tissue. **G** The co-localization of CCL17/CD68 in mice plaque tissues was detected by immunofluorescence. **H** The level of CD25 in the mice plaque was detected by immunofluorescence. * *p* < 0.05, ** *p* < 0.01, *** *p* < 0.001. *N* = 6

We knocked down the expression level of SENP3 in AS model mice. The findings revealed that sh-SENP3 could observably reduce SENP3 protein expression in the plaque tissue of AS mice (Fig. 6 A). Knocking down SENP3 could significantly reduce the arterial injury and plaque area of AS mice, and at the same time reduce the contents of the blood lipid levels (Fig. 6B and 6 C). Similarly, knocking down SENP3 reduced the level of inflammatory factors in arterial tissue of mice (Fig. 6D) and increased the expression of lipid-related genes (FASN, CD36, SCD1 and SREBP1) (Fig. 6E). Down-regulation of SENP3 can significantly increase the proportion of Treg cells (FOXP3, CD25) in CD4 cells (Fig. 6 F). Finally, we detected the levels of CCL17, CD68 and CD25 in plaque tissues. The results displayed that inhibition of SENP3 could reduce the levels of CCL17 and CD68 and increase the expression of CD25 (Fig. 6G and 6H). Similarly, in in vitro experiment, it was found that in the macrophages stimulated by ox-LDL, after simultaneously silencing SENP3 and adding 2-D08 (an inhibitor of SUMOylation), it was observed that CCL17 decreased after silencing SENP3, while the addition of 2-D08 partially reversed this effect (Fig. S1A and S1B). To sum up, our results show that SENP3 is involved in regulating the progress of AS.

## Discussion

**Fig. 7:**
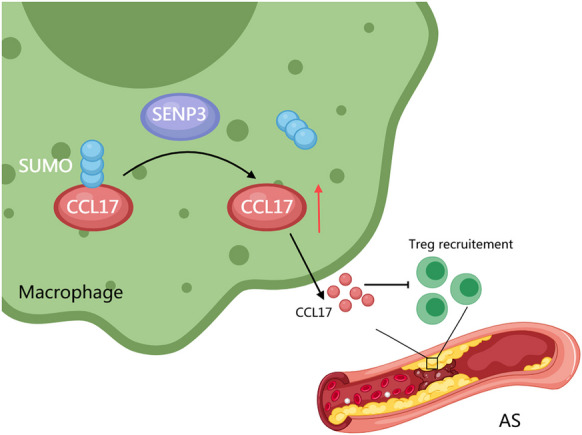
SENP3-mediated regulation of CCL17 in atherosclerosis. In atherosclerosis, SENP3 levels are elevated in macrophages, where it mediates the DeSUMOylation of CCL17, stabilizing its protein levels. Increased CCL17 secretion subsequently competitively inhibits Treg cell recruitment, exacerbating atherosclerosis progression

As a chronic multifactorial disease, atherosclerosis is characterized with inflammation and lipids accumulation in vessel walls(Bäck et al. 2019). Targeting the dyslipidemia and inflammation are promising methods for prevention and treatment of AS. It has been found that the high level of CCL17 in human serum is related to the high risk of cardiovascular diseases, and studies in mice show that CCL17 has pro-inflammatory effects in AS and colitis (Doring et al. 2024). Our study also confirmed that CCL17 could regulate the progress of AS, and further clarified that inhibiting CCL17 in macrophages improved AS by mediating the recruitment of Treg (Fig. 7).

CCL17 is involved in mediating or improving broad-spectrum immune response (Heiseke et al. 2012). As a member of chemokine family, CCL17 is expressed in many cells and is considered as a biomarker of disease severity (Landheer et al. 2014). Currently, the study on CCL17 mostly concentrates upon the promotion of AS by the mechanism of T cell dependence (Ye et al. 2015). Recent studies have found that macrophages expressing CCL17 can regulate immune microenvironment by secreting CCL17 (Zhang et al. 2021). Consistent with our research, we can also improve the pathological damage of AS mice by silencing CCL17 from macrophages. Studies have confirmed that there is a ligand-receptor relationship between CCL17 and CCR4 (Li et al. 2021). CCL17 is mainly involved in the recruitment of Th2 cells and CD4%2B Tregs by binding to CCR4 (Deng et al. 2021). Nowadays, it has been found that CCL22 can be used as the second ligand of CCR4, and CCL22 is related to macrophage activation, which is called macrophage-derived chemokine (Yogo et al. 2009). An interesting study found that CCL17 and CCL22 can participate in the regulation of Treg chemotaxis as competitive biased ligands of CCR4 in myocardial infarction. CCL17 mainly activates Gq signal downstream of CCR4, while CCL22 can simultaneously activate Gq and β-arrestin signal, and CCL17 competitively inhibits β-arrestin signal transduction and Treg chemotaxis stimulated by CCL22 (Feng et al. 2022). Therefore, CCL17 and CCL22 may have opposite regulation on diseases. Unlike acute myocardial infarction, where CCL17/CCL22 competition occurs transiently during acute inflammation, our study reveals that this chemokine interplay also exists in atherosclerosis, suggesting a chronic and persistent regulatory mechanism in immune cell recruitment and plaque progression. It has been reported that increasing the expression of CCL22 in human monocytes can promote the formation of AS in mice (Kimura et al. 2015). M2 macrophages can secrete CCL12, which affects the distribution of vascular smooth muscle cells and promotes the progress of AS (Kimura et al. 2018). Our research also revealed CCL22 expression was increased in AS model and co-located with macrophages (CD68). However, at present, there is still little research on CCL12 in cardiovAScular diseases, especially as. Our follow-up study also needs to further confirm whether CCL22 has the same or opposite regulatory mechanism as CCL17 in cardiovascular diseases.

Our study on the regulatory mechanism of CCL17 confirmed that its expression was enhanced by SENP3-mediated desumoylation modification in macrophages. SUMOylation is a post-translational modification of lysine residues. It is reported that the DeSUMOylation of GPR120 at K32 in vascular smooth muscle cells is related to the severity of AS (Yan et al. 2022). Up-regulating SUMO1 and increasing the SUMOylation of SERCA2a can alleviate vascular injury and thus protect as induced by diabetes (Liu et al. 2024). By overexpressing DeSUMOylation regulatory enzyme SENP3 in macrophages, we found that SENP3 can significantly increase the stability and expression of CCL17 protein. Further experiments confirmed that no DeSUMOylation was detected in CCL17 after K115R mutation, which suggested that K115 might be the main site of SUMOylation in CCL17. In consistent with our study, SENP3 was proved to induce M1 polarization and aggravated inflammation in different mechanic manners and aggravated inflammation. He et al. found that in LPS mediated acute lung injury, SENP3 induced M1 polarization via HIF-1α (He et al. 2023). Another study illustrated that SENP3 deficiency induced M2 polarization via Akt Akt1 deSUMOylation in breast cancer (Xiao et al. 2022). studies have also shown that SENP3 promotes ferroptosis of M2 macrophages and reduces the proportion of M2 macrophages by interacting with FSP1 and causing it to undergo SUMOylation, thereby regulating macrophage function and inflammatory response (Chen et al. 2024b). On the contrary, researcher also found SENP3 showed anti-inflammatory effect. For instance, by deSUMOylating NLRP3, SENP3 alleviated form cell formation (Chen et al. 2024a). In term of cardiovascular disease, SENP3 has been shown to be upregulated in atherosclerosis, where it stabilizes β-catenin protein through deSUMOylation, thereby promoting VSMC migration and aggravating atherosclerosis (Cai et al. 2021). Our study also found that SENP3 exacerbates atherosclerosis through a novel mechanism involving the deSUMOylation of CCL17.

A limitation of the present study is that only the ApoE^−/−^ mouse model was employed to investigate the role of SENP3-mediated DeSUMOylation of CCL17 in atherosclerosis. Although the ApoE −/− mouse is a classical and widely used model that recapitulates many key features of human AS, the use of a single model may restrict the generalizability of the findings. In future work, we plan to extend our validation to additional systems, including LDLR^−/−^ mice, PCSK9-induced hyperlipidemia models, diet-induced AS models, and ultimately clinical samples, to further strengthen the robustness and translational relevance of our results.

## Conclusion

To summarize, we found that the expression of CCL17 increased in AS mice and macrophages induced by oxLDL. CCL17 in macrophages is regulated by de-sunoylation mediated by SENP3, thus mediating the recruitment of Treg. At the same time, we also found that macrophages stimulated by oxLDL affect the chemotaxis of Treg through CCL17 and CCL22 competition. Our findings provide a new regulatory mechanism for AS progress.

## Supplementary Information

Below is the link to the electronic supplementary material.

ESM1 (PDF. 5.06 MB)

## Data Availability

The datasets used or analyzed during the current study are available from the corresponding author on reasonable request.

## Abbreviations

AAV: Adeno-associated virus
AS: Atherosclerosis
CCL17: C-C chemokine ligand 17
EC: Cholesteryl ester
FC: Free cholesterol
HE: Hematoxylin-eosin
HFD: High-fat diet
IL-1β: Interleukin-1β
LDL-C: Low-Density Lipoprotein Cholesterol
ox-LDL: Oxidized low-density lipoprotein
SUMOs: Small ubiquitin-like modifiers
SENPs: Sentrin/SUMO-specific protease
SPF: Special pathogen-free
TNF-α: Tumor necrosis factor-α
TC: Total cholesterol
TG: Triglycerides
VCAM-1: Vascular adhesion molecule-1

## References

[CR1] Bäck M, Yurdagul A Jr, Tabas I, Öörni K, Kovanen PT. Inflammation and its resolution in atherosclerosis: mediators and therapeutic opportunities. Nat Rev Cardiol. 2019;16:389–406. 10.1038/s41569-019-0169-2. 30846875 10.1038/s41569-019-0169-2 PMC6727648

[CR2] Bakogiannis C, Sachse M, Stamatelopoulos K, Stellos K. Platelet-derived chemokines in inflammation and atherosclerosis. Cytokine. 2019;122:154157. 10.1016/j.cyto.2017.09.013. 29198385 10.1016/j.cyto.2017.09.013

[CR3] Cai Z, Wang Z, Yuan R, Cui M, Lao Y, Wang Y, et al. Redox-sensitive enzyme SENP3 mediates vascular remodeling via de-SUMOylation of β-catenin and regulation of its stability. EBioMedicine. 2021;67:103386. 10.1016/j.ebiom.2021.103386. 34000626 10.1016/j.ebiom.2021.103386 PMC8138600

[CR4] Chen J, Sun X, Liu Y, Zhang Y, Zhao M, Shao L. Senp3 attenuates foam cell formation by desUMOylating Nlrp3 in macrophages stimulated with ox-LDL. Cell Signal. 2024a;117:111092. 10.1016/j.cellsig.2024.111092. 38331013 10.1016/j.cellsig.2024.111092

[CR5] Chen X, Wang J, Yang P, Liu HY, Zhong S, Lu C, et al. Senp3 sensitizes macrophages to ferroptosis via de-SUMOylation of FSP1. Redox Biol. 2024b;75:103267. 10.1016/j.redox.2024.103267. 39025016 10.1016/j.redox.2024.103267 PMC11301343

[CR6] Cohen Tervaert JW. Cardiovascular disease due to accelerated atherosclerosis in systemic vasculitides. Best Pract Res Clin Rheumatol. 2013;27:33–44. 10.1016/j.berh.2012.12.004. 23507055 10.1016/j.berh.2012.12.004

[CR7] Conaghan PG, Cook AD, Hamilton JA, Tak PP. Therapeutic options for targeting inflammatory osteoarthritis pain. Nat Rev Rheumatol. 2019;15:355–63. 10.1038/s41584-019-0221-y. 31068673 10.1038/s41584-019-0221-y

[CR8] Deng S, Jin P, Sherchan P, Liu S, Cui Y, Huang L, et al. Recombinant CCL17-dependent CCR4 activation alleviates neuroinflammation and neuronal apoptosis through the PI3K/AKT/Foxo1 signaling pathway after ICH in mice. J Neuroinflammation. 2021;18:62. 10.1186/s12974-021-02112-3. 33648537 10.1186/s12974-021-02112-3 PMC7923481

[CR9] Doring Y, van der Vorst EPC, Yan Y, Neideck C, Blanchet X, Jansen Y, et al. Identification of a non-canonical chemokine-receptor pathway suppressing regulatory T cells to drive atherosclerosis. Nat Cardiovasc Res. 2024;3:221–42. 10.1038/s44161-023-00413-9. 39044999 10.1038/s44161-023-00413-9 PMC7616283

[CR10] Feng G, Bajpai G, Ma P, Koenig A, Bredemeyer A, Lokshina I, et al. Ccl17 aggravates myocardial injury by suppressing recruitment of regulatory T cells. Circulation. 2022;145:765–82. 10.1161/CIRCULATIONAHA.121.055888. 35113652 10.1161/CIRCULATIONAHA.121.055888 PMC8957788

[CR11] Gencer S, Evans BR, van der Vorst EPC, Doring Y, Weber C. Inflammatory chemokines in atherosclerosis. Cells. 2021. 10.3390/cells10020226. 33503867 10.3390/cells10020226 PMC7911854

[CR12] Hamilton JA. Gm-csf-dependent inflammatory pathways. Front Immunol. 2019;10:2055. 10.3389/fimmu.2019.02055. 31552022 10.3389/fimmu.2019.02055 PMC6737278

[CR13] Hannawi S, Hannawi H, Al Salmi I. Cardiovascular disease and subclinical atherosclerosis in rheumatoid arthritis. Hypertens Res. 2020;43:982–4. 10.1038/s41440-020-0483-4. 32483312 10.1038/s41440-020-0483-4

[CR14] He S, Fan C, Ji Y, Su Q, Zhao F, Xie C, Chen X, Zhang Y, Chen Y. Senp3 facilitates M1 macrophage polarization via the HIF-1alpha/PKM2 axis in lipopolysaccharide-induced acute lung injury. Innate Immun. 2023;29:25–34. 10.1177/17534259231166212. 37016838 10.1177/17534259231166212 PMC10164277

[CR15] Heiseke AF, Faul AC, Lehr HA, Forster I, Schmid RM, Krug AB, et al. CCL17 promotes intestinal inflammation in mice and counteracts regulatory T cell-mediated protection from colitis. Gastroenterology. 2012;142:335–45. 10.1053/j.gastro.2011.10.027. 22057112 10.1053/j.gastro.2011.10.027

[CR16] Inzucchi SE, Claggett BL, Vaduganathan M, Desai AS, Jhund PS, de Boer RA, et al. Efficacy and safety of dapagliflozin in patients with heart failure with mildly reduced or preserved ejection fraction by baseline glycaemic status (DELIVER): a subgroup analysis from an international, multicentre, double-blind, randomised, placebo-controlled trial. Lancet Diabetes Endocrinol. 2022;10:869–81. 10.1016/S2213-8587(22)00308-4. 36372069 10.1016/S2213-8587(22)00308-4

[CR17] Kimura S, Wang KY, Yamada S, Guo X, Nabeshima A, Noguchi H, et al. CCL22/macrophage-derived chemokine expression in apolipoprotein E-deficient mice and effects of histamine in the setting of atherosclerosis. J Atheroscler Thromb. 2015;22:599–609. 10.5551/jat.27417. 25492567 10.5551/jat.27417

[CR18] Kimura S, Noguchi H, Nanbu U, Wang KY, Sasaguri Y, Nakayama T. Relationship between CCL22 expression by vascular smooth muscle cells and macrophage histamine receptors in atherosclerosis. J Atheroscler Thromb. 2018;25:1240–54. 10.5551/jat.44297. 29794410 10.5551/jat.44297 PMC6249366

[CR19] Kumar A, Zhang KY. Advances in the development of SUMO specific protease (SENP) inhibitors. Comput Struct Biotechnol J. 2015;13:204–11. 10.1016/j.csbj.2015.03.001. 25893082 10.1016/j.csbj.2015.03.001 PMC4397505

[CR20] Landheer J, de Bruin-Weller M, Boonacker C, Hijnen D, Bruijnzeel-Koomen C, Rockmann H. Utility of serum thymus and activation-regulated chemokine as a biomarker for monitoring of atopic dermatitis severity. J Am Acad Dermatol. 2014;71(6):1160–6. 10.1016/j.jaad.2014.07.031. 25199679 10.1016/j.jaad.2014.07.031

[CR21] Lee KM, Jarnicki A, Achuthan A, Fleetwood AJ, Anderson GP, Ellson C, et al. CCL17 in inflammation and pain. J Immunol. 2020;205:213–22. 10.4049/jimmunol.2000315. 32461237 10.4049/jimmunol.2000315

[CR22] Li H, Wang C, Li X, Kong Y, Sun W. CCL17-CCR4 axis contributes to the onset of vitiligo in mice. Immunity Inflamm Dis. 2021;9:702–9. 10.1002/iid3.423. 10.1002/iid3.423 PMC8342221 34077992

[CR23] Libby P. The changing landscape of atherosclerosis. Nature. 2021;592:524–33. 10.1038/s41586-021-03392-8. 33883728 10.1038/s41586-021-03392-8

[CR24] Liu J, Xu S, Gao B, Yuan M, Zhong L, Guo R. Protective effect of SERCA2a-SUMOylation by SUMO-1 on diabetes-induced atherosclerosis and aortic vascular injury. Mol Cell Biochem. 2024. 10.1007/s11010-024-04953-x. 38438822 10.1007/s11010-024-04953-x

[CR25] Pedro-Botet J, Climent E, Benaiges D. Atherosclerosis and inflammation. New therapeutic approaches. Med Clin (Barc). 2020;155:256–62. 10.1016/j.medcli.2020.04.024 10.1016/j.medcli.2020.04.024 32571617

[CR26] Tabares-Guevara JH, Villa-Pulgarin JA, Hernandez JC. Atherosclerosis: immunopathogenesis and strategies for immunotherapy. Immunotherapy. 2021;13:1231–44. 10.2217/imt-2021-0009. 34382409 10.2217/imt-2021-0009

[CR27] Talamillo A, Ajuria L, Grillo M, Barroso-Gomila O, Mayor U, Barrio R. Sumoylation in the control of cholesterol homeostasis. Open Biol. 2020;10:200054. 10.1098/rsob.200054. 32370667 10.1098/rsob.200054 PMC7276529

[CR28] van der Vorst EP, Doring Y, Weber C. Chemokines. Arterioscler Thromb Vasc Biol. 2015;35:e52–6. 10.1161/ATVBAHA.115.306359. 26490276 10.1161/ATVBAHA.115.306359

[CR29] Vilgelm AE, Richmond A. Chemokines modulate immune surveillance in tumorigenesis, metastasis, and response to immunotherapy. Front Immunol. 2019;10:333. 10.3389/fimmu.2019.00333. 30873179 10.3389/fimmu.2019.00333 PMC6400988

[CR30] Weber C, Meiler S, Doring Y, Koch M, Drechsler M, Megens RT, et al. CCL17-expressing dendritic cells drive atherosclerosis by restraining regulatory T cell homeostasis in mice. J Clin Invest. 2011;121:2898–910. 10.1172/JCI44925. 21633167 10.1172/JCI44925 PMC3223829

[CR31] Witty J, Aguilar-Martinez E, Sharrocks AD. SENP1 participates in the dynamic regulation of Elk-1 SUMOylation. Biochem J. 2010;428:247–54. 10.1042/BJ20091948. 20337593 10.1042/BJ20091948 PMC2943748

[CR32] Xiao M, Bian Q, Lao Y, Yi J, Sun X, Sun X, et al. Senp3 loss promotes M2 macrophage polarization and breast cancer progression. Mol Oncol. 2022;16:1026–44. 10.1002/1878-0261.12967. 33932085 10.1002/1878-0261.12967 PMC8847990

[CR33] Yan CH, Liu HW, Tian XX, Li J, Ding Y, Li Y, et al. AMPKα2 controls the anti-atherosclerotic effects of fish oils by modulating the SUMOylation of GPR120. Nat Commun. 2022;13(1):7721. 10.1038/s41467-022-34996-x. 36513627 10.1038/s41467-022-34996-x PMC9747961

[CR34] Ye Y, Yang X, Zhao X, Chen L, Xie H, Zeng Y, et al. Serum chemokine CCL17/thymus activation and regulated chemokine is correlated with coronary artery diseases. Atherosclerosis. 2015;238:365–9. 10.1016/j.atherosclerosis.2014.12.047. 25555269 10.1016/j.atherosclerosis.2014.12.047

[CR35] Yogo Y, Fujishima S, Inoue T, Saito F, Shiomi T, Yamaguchi K, et al. Macrophage derived chemokine (CCL22), thymus and activation-regulated chemokine (CCL17), and CCR4 in idiopathic pulmonary fibrosis. Respir Res. 2009;10:80. 10.1186/1465-9921-10-80. 19715610 10.1186/1465-9921-10-80 PMC2741459

[CR36] Zhang A, Xu Y, Xu H, Ren J, Meng T, Ni Y, et al. Lactate-induced M2 polarization of tumor-associated macrophages promotes the invasion of pituitary adenoma by secreting CCL17. Theranostics. 2021;11:3839–52. 10.7150/thno.53749. 33664865 10.7150/thno.53749 PMC7914368

[CR37] Zhu Y, Xian X, Wang Z, Bi Y, Chen Q, Han X, et al. Research progress on the relationship between atherosclerosis and inflammation. Biomolecules. 2018;8:80. 10.3390/biom8030080. 30142970 10.3390/biom8030080 PMC6163673

