# Supplementary material for: SENP3 mediated DeSUMOylation of macrophage derived CCL17 accelerates atherosclerosis via regulation of Treg
Source: Cell Biol Toxicol. 2025 Nov 21;41(1):151. doi: 10.1007/s10565-025-10099-3 (PMC12634754; doi:10.1007/s10565-025-10099-3)

Fig2A

CCL17

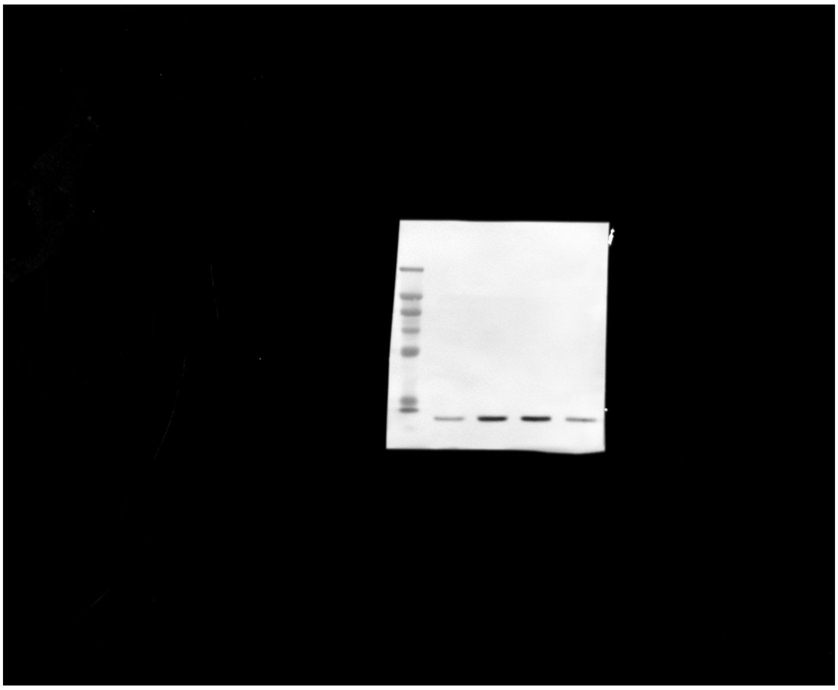

β -actin

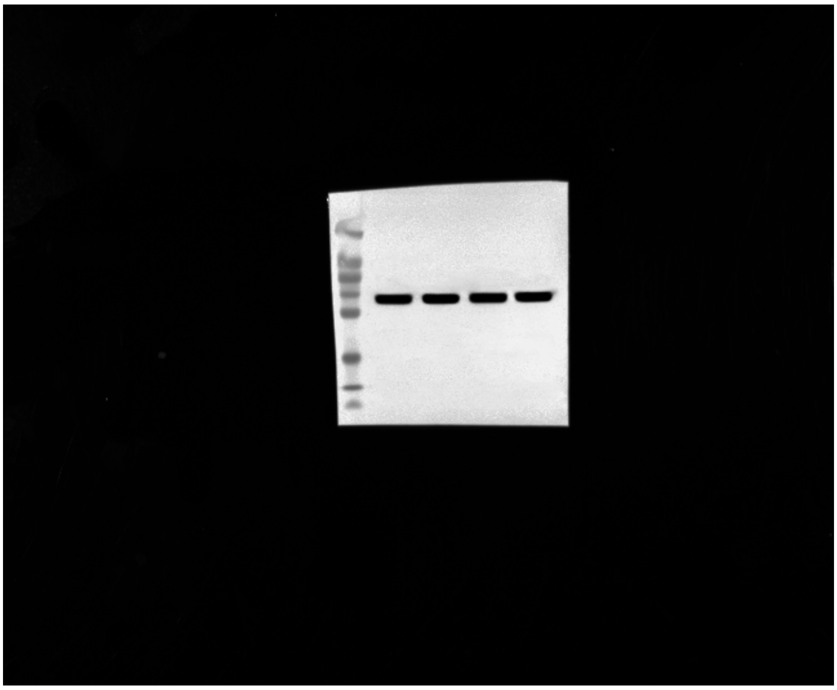

FIG5A

SENP3

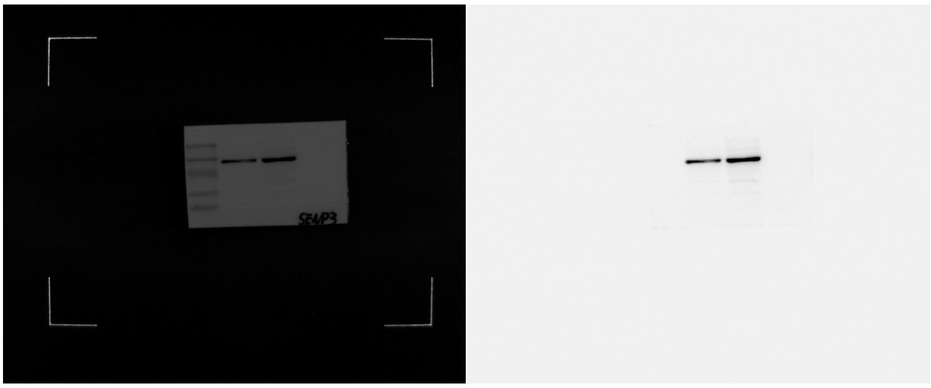


β -actin

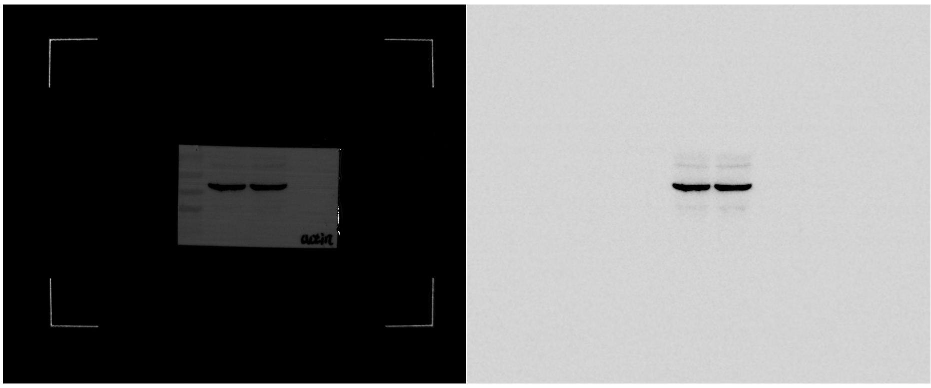

FIG 5B

CCL17

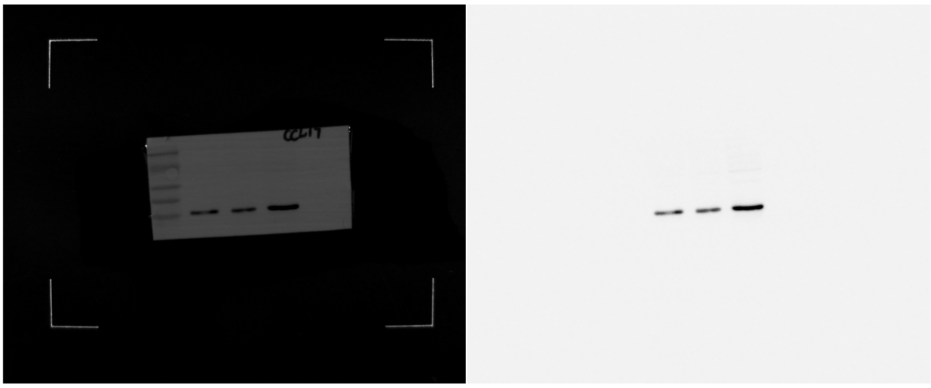

SENP3

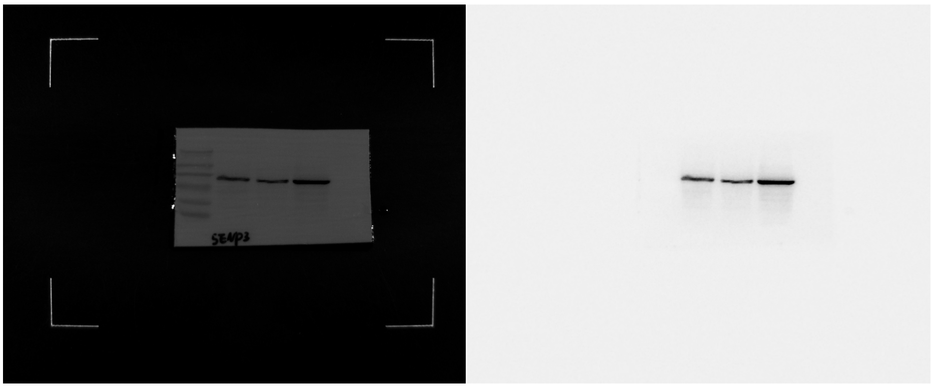

β -actin

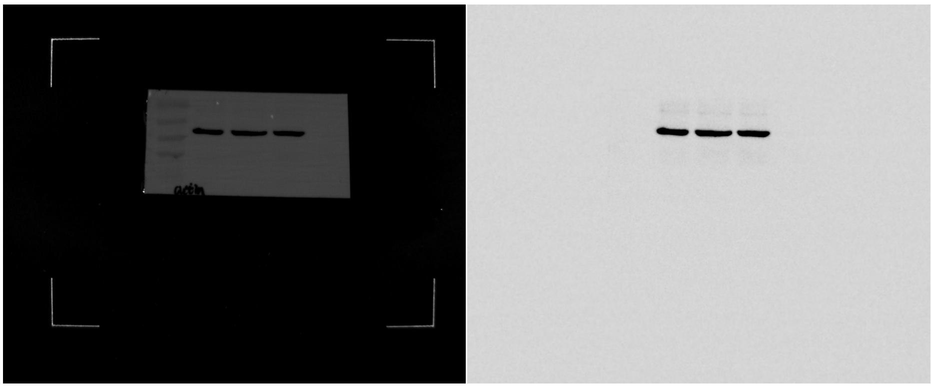

FIG 5D

CCL17

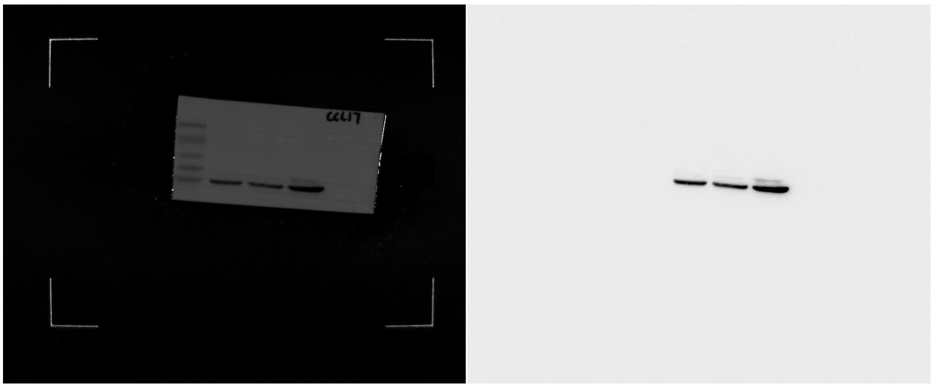


SUMO1

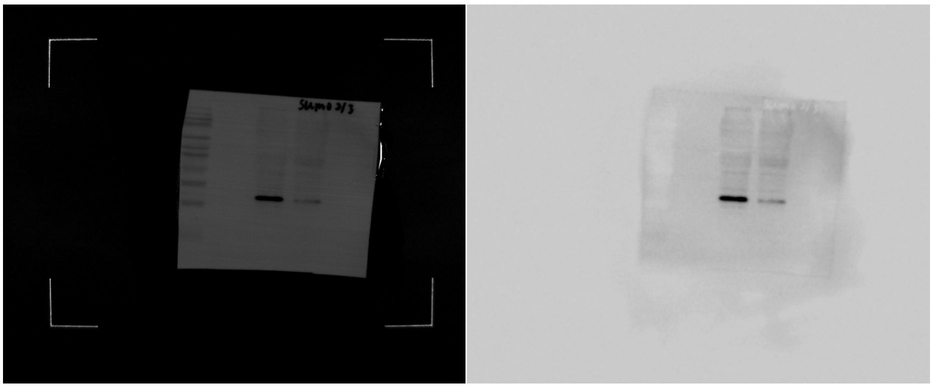

β -actin

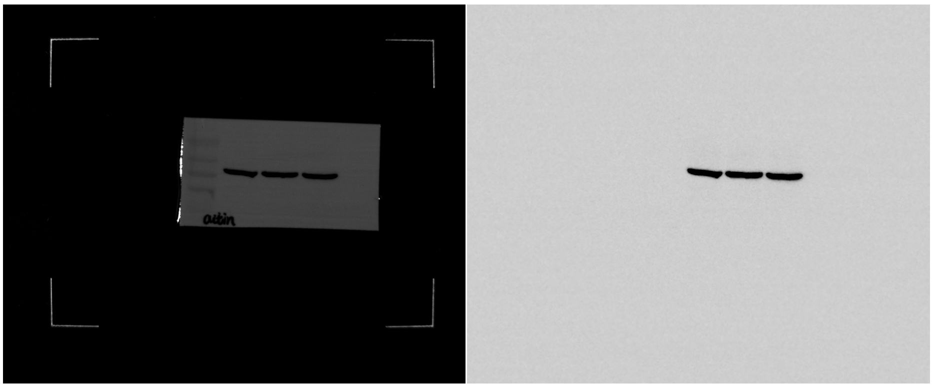

FIG 5E

CCL17

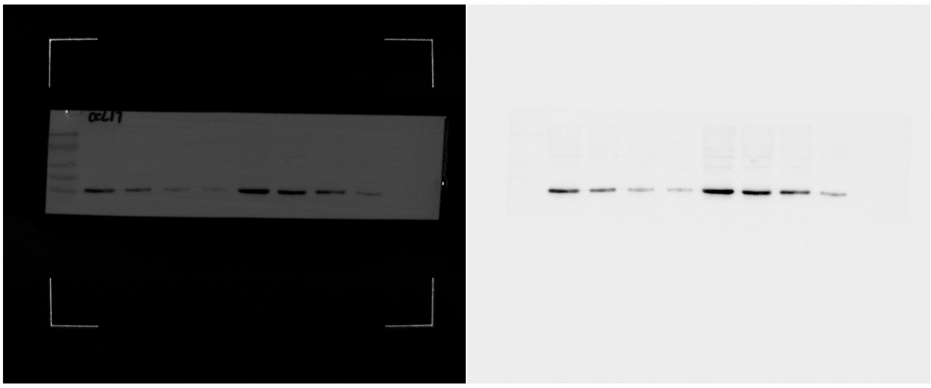

β -actin

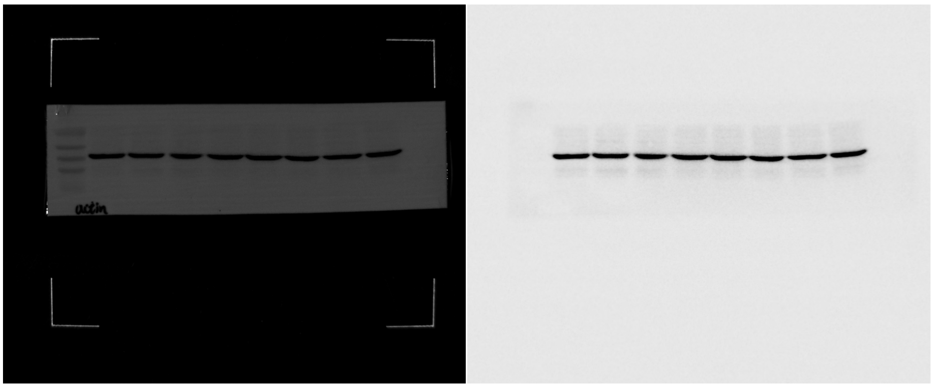

FIG 5G

luput

CCL17

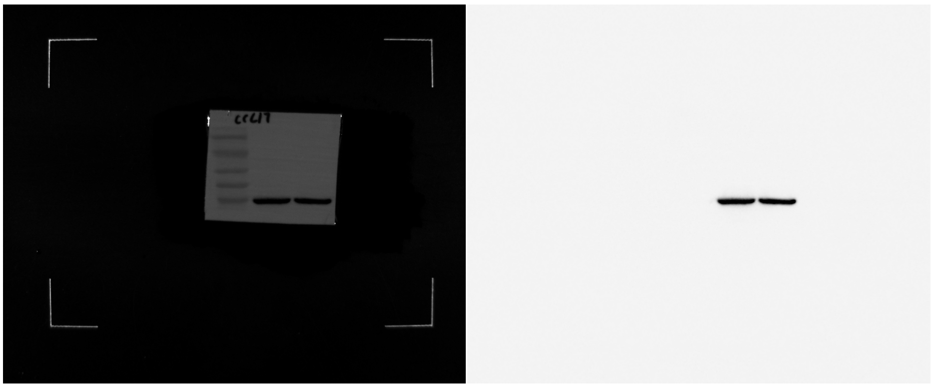

SENP3

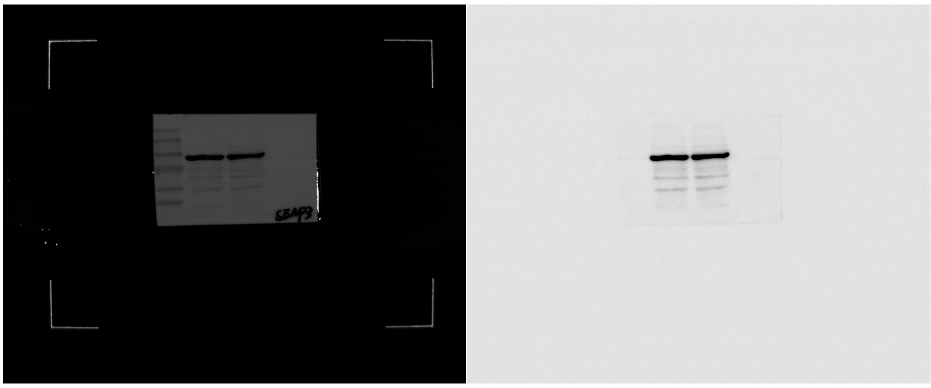


β -actin

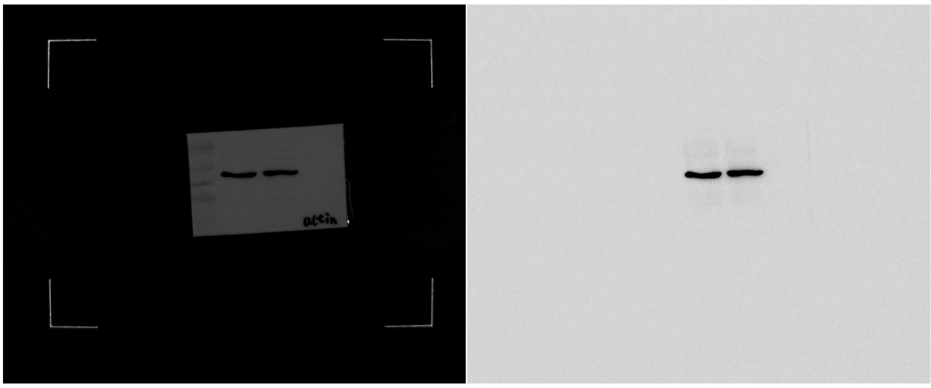

IP

CCL17

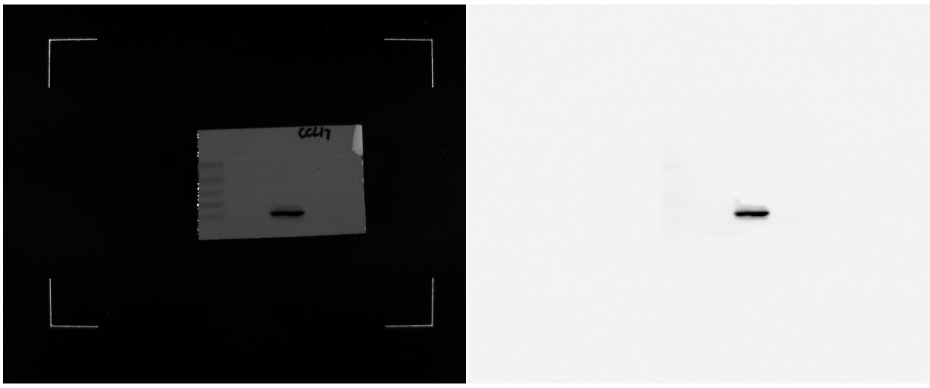

SENP3

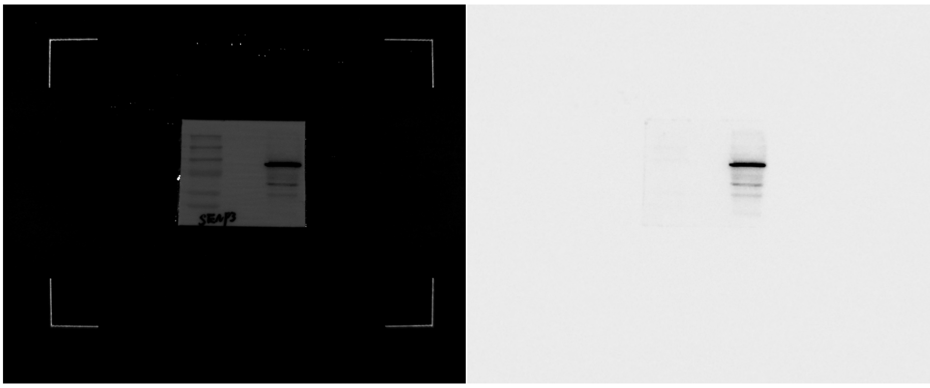

FIG 6A

SENP3

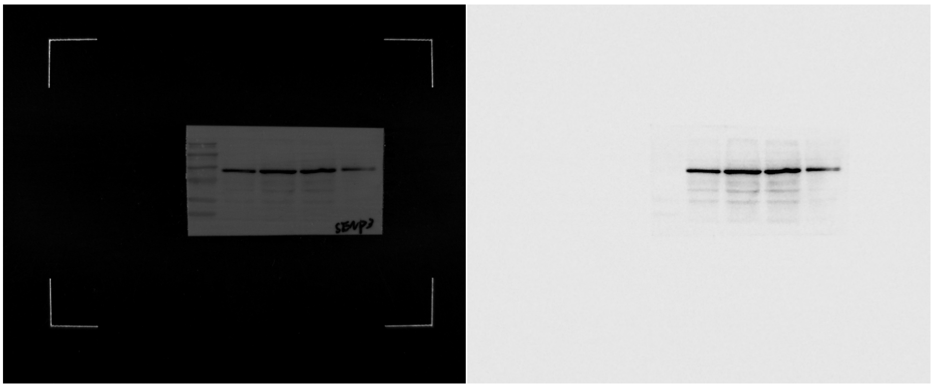


β -actin

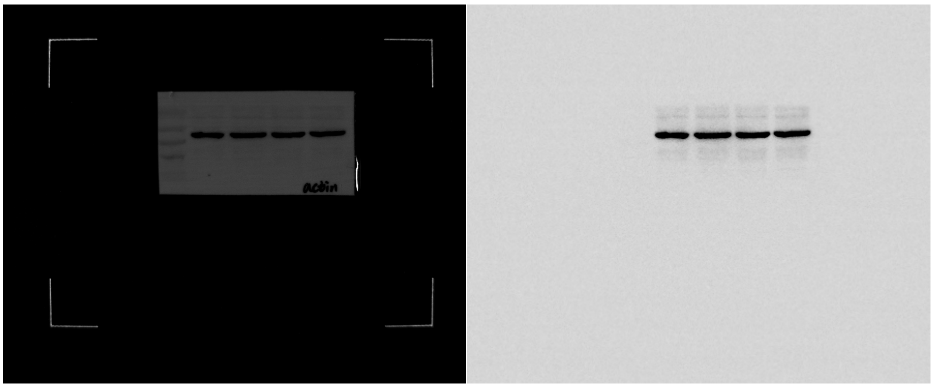

Supplement: Supplementary file 1 — (PDF. 5.06 MB) [file 10565_2025_10099_MOESM1_ESM.pdf]
